# Polymers Enhance Chlortetracycline Hydrochloride Solubility

**DOI:** 10.3390/ijms251910591

**Published:** 2024-10-01

**Authors:** Chao Zhang, Bing Li, Yubin Bai, Yangling Liu, Yong Zhang, Jiyu Zhang

**Affiliations:** 1 College of Veterinary Medicine, Gansu Agricultural University, Anning District, Lanzhou 730070, China; zchao0901@163.com (C.Z.); 18215190739@163.com (Y.L.); 2 Lanzhou Institute of Husbandry and Pharmaceutical Sciences, Chinese Academy of Agricultural Sciences, Qilihe District, Lanzhou 730050, China; pharm2005bl@126.com (B.L.); baiyb1011@163.com (Y.B.)

**Keywords:** chlortetracycline hydrochloride, solid dispersion, solubility, povidone K30, hydroxypropyl-β-cyclodextrin, gelatin, characterization

## Abstract

Chlortetracycline hydrochloride (CTC) is a broad-spectrum tetracycline antibiotic with a wide range of antibacterial activities. Due to low solubility, poor stability, and low bioavailability, clinical preparation development is limited. We sought to improve these solubility and dissolution rates by preparing solid dispersions. A hydrophilic polymer was selected as the carrier, and a solid dispersion was prepared using a medium grinding method, with samples characterized by scanning electron microscopy (SEM), differential scanning calorimetry (DSC), thermogravimetric analysis (TGA), powder X-ray diffraction (PXRD), Fourier-transform infrared spectroscopy (FT-IR), and particle size distribution (PSD). To maximize CTC solubility and stability, different polymer types and optimal drug-to-polymer ratios were screened. The solubility of optimized povidone K30 (PVPK30) (1/0.75, *w*/*w*)-, hydroxypropyl-β-cyclodextrin (HP-β-CD) (1/2, *w*/*w*)-, and gelatin (1/1, *w*/*w*)-based solid dispersions was 6.25-, 7.7-, and 3.75-fold higher than that of pure CTC powder, respectively. Additionally, in vitro dissolution studies showed that the gelatin-based solid dispersion had a higher initial dissolution rate. SEM and PS analyses confirmed that this dispersion had smaller and more uniform particles than PVPK30 and HP-β-CD dispersions. Therefore, successful solid polymer dispersion preparations improved the CTC solubility, dissolution rates, and stability, which may have potential as drug delivery systems.

## 1. Introduction

**Figure 1:**
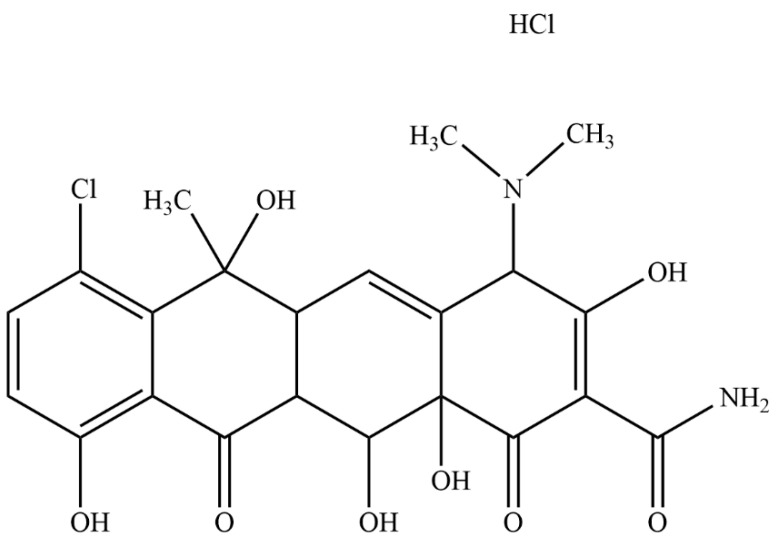
Chemical structure of chlortetracycline hydrochloride (CTC).

Chlortetracycline hydrochloride (CTC, Figure 1) (6-methyl-4-(dimethylamino)-3,6,10,12,12α-pentahydroxy-1,1-dioxo-7-chloro-1,4,4α,5,5α,6,11,12α-octahydro-2-tetrabenamide hydrochloride) is a broad-spectrum antibiotic of the tetracycline family [1], produced by the genus Actinomyces. As CTC acts by inhibiting protein synthesis in sensitive microorganisms, it has wide antibacterial activity ranges against both Gram-positive and Gram-negative bacteria and is widely used to treat animal and human infectious diseases [2,3,4]. CTC is a class II drug (Biopharmaceutical Classification System), and its low aqueous solubility (approximately 4 mg/mL) is associated with low oral bioavailability [5], which limits clinical applications. Therefore, an effective and appropriate CTC drug delivery system is required to address these shortcomings.

Many different solubilization technologies have been studied, including physical and chemical modification methods, such as cyclodextrin inclusion complexes [6], nanosuspensions [7], and salt/eutectic [8] and solid [9,10,11] dispersions. Of the various technologies, amorphous solid dispersion [12] effectively improves drug solubility and bioavailability by reducing the drug particle size (PS) and improving wettability, and it has higher drug loading and stability. This technology is widely used to convert drugs from crystalline to amorphous forms [13]. When compared with crystalline forms, amorphous forms significantly improve solubility and dissolution rates, as evidenced by the literature [14]. However, as amorphous forms are in a physically unstable high-energy state, this greatly affects drug stability [15]. To overcome such issues, polymers have shown promise as crystallization inhibitors, with some studies proposing that polymers generate long-term supersaturation by reducing drug molecule mobility [16]. As polymers with different molecular weights have different solubilization effects on insoluble drugs and also on the crystallization inhibition degree, the correct polymers must be selected for insoluble drugs.

Povidone K30 (PVPK30) is a common solid-dispersion carrier; its carbonyl oxygen interacts with the hydrogen-bond forming molecules in a drug, which inhibits amorphous drug crystallization. PVPK30’s effects on drug stability have been widely reported [17,18]. Similarly, both hydroxypropyl-β-cyclodextrin (HP-β-CD) and gelatin have also been widely used in drug research due to their water solubility, non-toxicity, and excellent biocompatibility traits [19,20,21].

The aim of this study is to prepare a new solid drug–polymer dispersion using medium ball milling to improve CTC solubility and stability. Based on solubility studies, the optimal polymer type and drug/polymer ratio are selected. Finally, the solid dispersion’s physical and chemical properties are analyzed.

## 2. Results and Discussion

### 2.1. Effects of Different Types of Polymers on the Solubility of CTC

**Figure 2:**
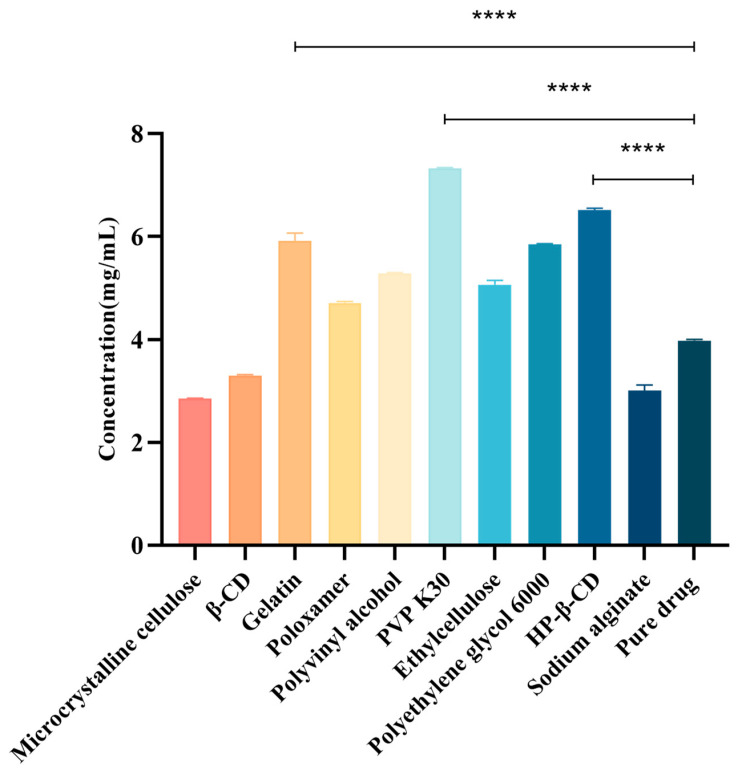
Different compounds’ effects on CTC at 1:1 mass ratios. **** *p*-Value < 0.0001.

The choice of the polymer carrier is crucial when determining insoluble drug solubility and how it directly affects drug stability and bioavailability [22]. Therefore, to select a suitable hydrophilic polymer as the CTC solid-dispersion carrier, we evaluated the effects of ten polymers on the CTC solubility. As shown (Figure 2), the polymers and CTC were determined at a 1:1 mass ratio. We observed that drug solubility increased with polymer addition. Of these polymers, PVPK30 significantly improved the CTC solubility; the solubility at 7.35 mg/mL was 1.84-fold higher than that of pure CTC (4 mg/mL), suggesting that interactions between PVPK30 and CTC disrupted the drug’s interactions with itself and thereby improved solubility. Also, HP-β-CD’s solubility effects toward CTC (6.55 mg/mL) were increased 1.64-fold, while gelatin’s solubility effects toward CTC (6.05 mg/mL) were increased approximately 1.51-fold. In contrast, microcrystalline cellulose, β-CD, and sodium alginate had no solubilizing effects toward CTC. Thus, polymer choice is a critical factor when improving insoluble CTC’s solubility. Therefore, the PVPK30, HP-β-CD, and gelatin carriers were selected for further research.

### 2.2. Polymer Concentration Effects on Solid-Dispersion Solubility in CTC

**Figure 3:**
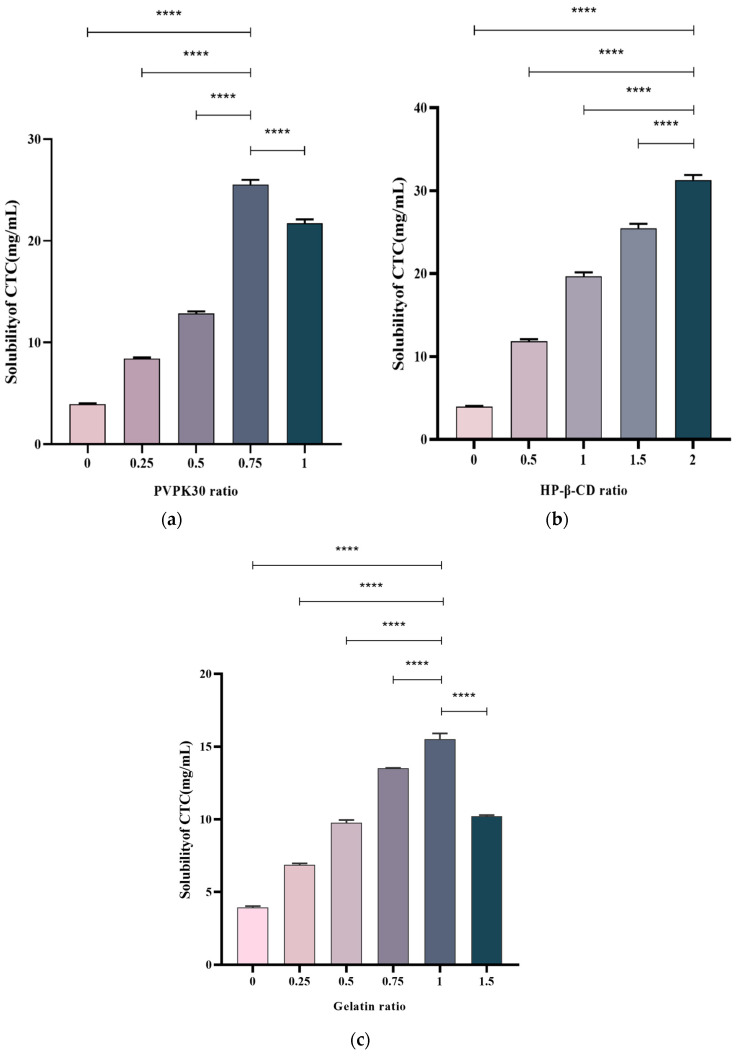
Polymer concentration effects on CTC-loaded solid-dispersion solubility. (**a**) PVPK30; (**b**) HP-β-CD; and (**c**) gelatin. **** *p*-Value < 0.0001.

The drug/polymer effects at different ratios on the CTC solubility were examined. Multiple ratios were selected to prepare solid CTC dispersions. PVPK30-based solid dispersions, with CTC-to-PVPK30 weight ratios of 1/0, 1/0.25, 1/0.5, and 1/1, were prepared using a medium grinding method. As observed (Figure 3a), with a gradual increase in the PVPK30 dose, the CTC solubility in solid dispersions significantly increased. In general, insoluble drugs’ solubility increases with an increase in water-soluble polymers [23]. However, when the CTC/PVPK30 ratio reached 1/0.75, the CTC solubility decreased as the PVPK30 quantity increased. This phenomenon also occurred in gelatin-based solid dispersions (Figure 3c). When the CTC-to-gelatin ratio reached 1/1, the CTC solubility decreased with an increased gelatin dose. These results show that the drug-to-polymer ratio was critical for improving drug solubility. Therefore, the PVPK30- (weight ratio = 1/0.75) and gelatin-based solid dispersions (weight ratio = 1/1) were selected for further study. Subsequently, HP-β-CD-based solid dispersions with 1/0, 1/0.5, 1/1, 1/1.5, and 1/2 CTC and HP-β-CD weight ratios were prepared using the medium grinding method. The CTC solubility (Figure 3b) increased with increasing HP-β-CD quantity. This observation was consistent with most solid drug solubility dispersions [24]. Therefore, we also selected HP-β-CD-based solid dispersions (weight ratio = 1/2) for further research. According to the above results, PVPK30-, HP-β-CD-, and gelatin-based solid dispersions improved the CTC solubility by approximately 6.25-, 7.69-, and 3.77-fold, respectively, when compared with pure CTC powder, indicating that optimal drug-to-polymer ratios, based on polymer type, were critical for maximizing the solid-dispersion systems.

### 2.3. In Vitro Dissolution Studies

**Figure 4:**
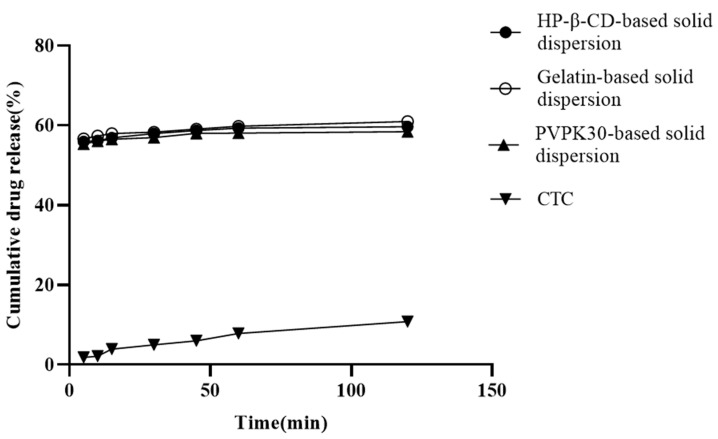
In vitro CTC-loaded solid-dispersion dissolution profiles.

**Figure 5:**
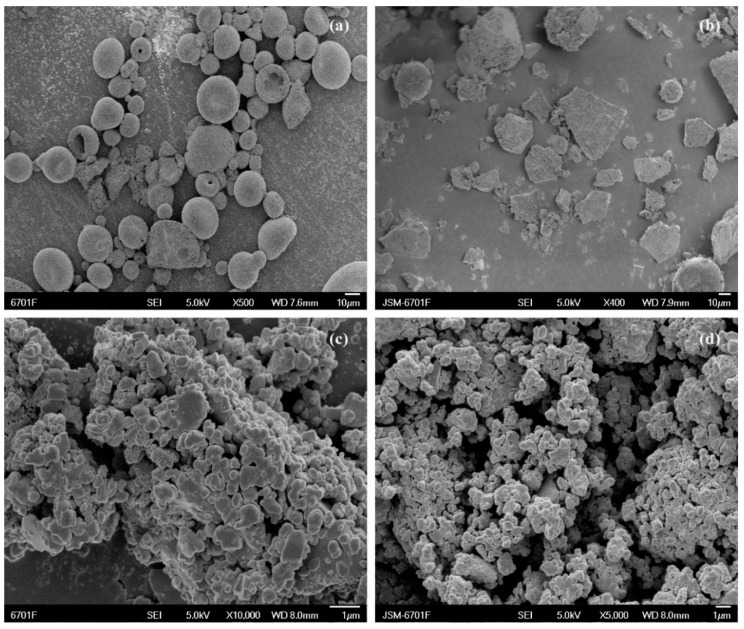
Scanning electron micrographs showing (**a**) CTC powder; (**b**) PVPK30-based solid dispersion; (**c**) HP-β-CD-based solid dispersion; and (**d**) gelatin-based solid dispersion.

In vitro release is a key factor affecting insoluble drug bioavailability during oral absorption [25]. Solid dispersions based on hydrophilic carries help improve the insoluble drugs’ wettability and reduce interfacial tension between the carrier and the dissolved medium [26]. The three solid dispersions prepared in this study are shown in Figure 4. At 120 min, the PVPK30-, HP-β-CD-, and gelatin-based solid dispersions’ dissolution rates were approximately 57.78%, 59.95%, and 60.89%, respectively. When compared with pure CTC (10.65%) and CTC premix [27], these dissolution rates were significantly higher, implying that the hydrophilic nature of pentaerythritol and the polymer may form a tight association with the CTC drug particles, causing partial amorphism and thus increasing drug dissolution. This results in increased wettability and dispersion of the drug. Additionally, the initial dissolution rates are also used to evaluate CTC’s dissolution in polymers. We observed that the gelatin-based solid dispersions’ dissolution rate in the first 30 min was significantly higher than that for both PVPK30- and HP-β-CD-based solid dispersions, indicating a higher initial wettability, which was possibly due to the smaller PS in gelatin-based solid dispersions. This result was consistent with the characterization results (Figure 5d). After 60 min, it was observed that the dissolution rates of all three solid dispersions were relatively slow, suggesting a potential final dissolution rate when compared to long-term saturation solubility results. Similarly, the release data were also used to study the release kinetics, and it was found that the release of CTC in the three solid dispersing regime agents followed the Peppas–Korsmeyer model, in which drug release followed the diffusion process.

### 2.4. Stability of CTC-Loaded Solid Dispersion

The results of the influencing-factor experiments on the solid dispersions including high temperature, high humidity, and strong light are shown in Table 1. The appearance of the three solid dispersions was yellow and did not change during the whole period of the influencing-factor experiments. In strong light, there was no significant difference in the content of PVPK30-based solid dispersions at 5 days and 10 days, which markedly enhanced the stability of CTC under illumination. Only 3.47% of the HP-β-CD-based solid dispersion was degraded after 10 days. The gelatin-based solid dispersion exhibited slight sensitivity, with degradation rates of 7.5% and 8.27% at 5 days and 10 days, respectively. No differences were noted in the content of the PVPK30- and HP-β-CD-based solid dispersions when subjected to high-temperature and high-humidity conditions. The gelatin-based solid dispersion showed mild sensitivity at 60 °C/75% ± 1%, resulting in degradation rates of 5.22% and 6.73% on days five and ten, respectively. Following an assessment of the influencing factors, all three types of solid dispersions complied with the standards set forth by the Chinese Veterinary Pharmacopoeia (labeled amount: 90–110%) [28] and demonstrated good stability.

### 2.5. Characterization

#### 2.5.1. Scanning Electron Microscopy (SEM)

SEM facilitates surface morphological observations and element composition analyses of different solid materials (e.g., PVPK30). SEM was used to examine the surface morphology of pure CTC and its complex (Figure 5 and Figure S1). Pure CTC had spherical structures (Figure 5a), while PVPK30 was composed of smooth surfaces and amorphous spherical particles (Figure S1e), while HP-β-CD showed spherical cavity structures (Figure S1f), and gelatin showed massive, irregular structures (Figure S1c). These surface morphologies were consistent with previous reports [29,30,31]. The physical mixture, simple M-SD prepared by fully mixing with CTC, showed a common CTC and polymer morphology (Figure S1c,e,f) without changing host and guest molecule morphologies during the process. In contrast, solid dispersions prepared by medium ball milling underwent drastic changes; amorphous forms were observed with disappearing spherical drug structures, suggesting that CTC was possibly amorphously distributed in the carrier. Additionally, gelatin-based solid dispersions exhibited smaller, more uniform particles that combined to form aggregates, indicating improved initial wettability and higher initial dissolution rates than the other solid dispersions.

#### 2.5.2. Particle Size Distributions (PSDs)

**Figure 6:**
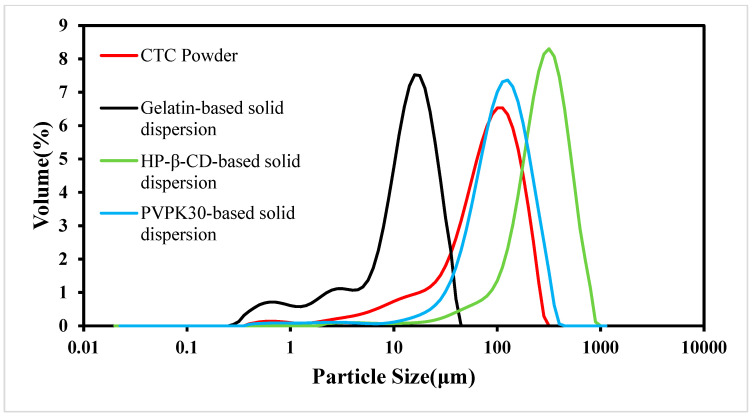
CTC and CTC-loaded solid-dispersion particle size distributions.

As the PS is an important factor affecting drug dissolution rates, solid dispersions must have uniform PSs and shapes [32]. In this study, we examined CTC PS distributions in the three solid dispersions (Figure 6). When compared with CTC powder, PS in the gelatin-based solid dispersions was significantly reduced. However, PS in the HP-β-CD- and PVPK30-based solid dispersions did not decrease when compared with that of the CTC powder, especially the PS in PVPK30-based solid dispersions, which increased significantly. This was because the PVPK30-based solid dispersion particles showed irregular bulk structures, resulting in wide PS distributions. In addition, the wettability of solid dispersions is also related to the improvement of the solubility of insoluble drugs [33]. The data show [34] that there is a direct correlation between the wettability of solid dispersions and the dissolution rate. Therefore, with an increase in the wettability of gelatin-based solid dispersions, the initial dissolution rate is higher than that of the HP-β-CD and PVPK30-based solid dispersions. These results were consistent with the SEM trends.

#### 2.5.3. Differential Scanning Calorimetry (DSC)

**Figure 7:**
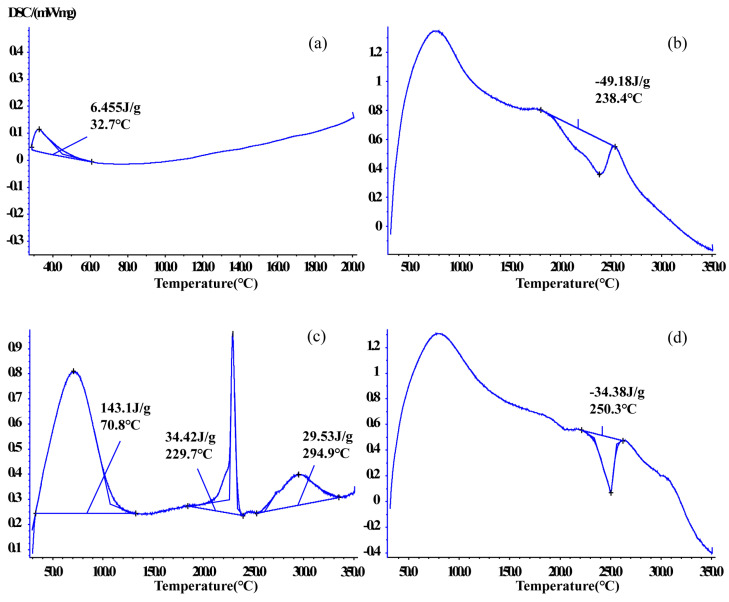
Comparing differential scanning calorimetric thermograms: (**a**) CTC powder; (**b**) PVPK30-based solid dispersion; (**c**) HP-β-CD-based solid dispersion; and (**d**) gelatin-based solid dispersion.

DSC is used to monitor interactions between host and guest molecules and shows melting point changes in drugs [35]. DSC was used to verify thermal behaviors in CTC and solid dispersions in this study (Figure 7 and Figure S2). The CTC powder DSC curve showed an endothermic peak at 32.7 °C. PVPK30 showed two broad endothermic peaks at 78.4 °C and 431.9 °C. The first spike was attributed to water molecule release and the second spike to PVPK30 decomposition. However, the physical mixture curve was dissimilar to that for PVPK30 and had a sharp exothermic peak at 249.8 °C (ΔH = −20.17 J/g), indicating a crystalline form.

The same phenomenon was observed for gelatin, which had two endothermic peaks at 221.9 °C and 268.5 °C and an exothermic peak at 244.1 °C. But the sample weight did not change. The physical mixture curve had a sharp exothermic peak at 250.2 °C (ΔH = −44.52 J/g).

The HP-β-CD physical mixture was different to the other mixtures; an endothermic peak was recorded at 70.4 °C, indicating water loss due to dehydration, consistent with previous reports [36]. The physical mixture also showed endothermic peaks at 72.5 °C, 238.3 °C, and 290.3 °C and an exothermic peak at 335.9 °C, indicating sample melting. In medium ball milling-prepared PVPK30- and gelatin-based solid dispersions (Figure 7b,d), the characteristic peaks relative to the drug disappeared, with exothermic peaks at 238.4 °C and 250.3 °C, respectively. The ΔH value (−34.38 J/g) of the gelatin-based solid dispersion was lower than that of the physical mixture (ΔH = −44.52 J/g), which indicated that CTC had partially replaced water binding in gelatin, resulting in physical interactions. The HP-β-CD solid dispersion contained three endothermic peaks at 70.8 °C, 229.7 °C, and 294.9 °C, with corresponding ΔH (34.42 J/g) and ΔH (29.53 J/g) values lower than those in the physical mixture. Additionally, characteristic peaks related to the CTC boiling point disappeared, further confirming enhanced CTC thermal stability.

#### 2.5.4. Thermogravimetric Analysis (TGA)

**Figure 8:**
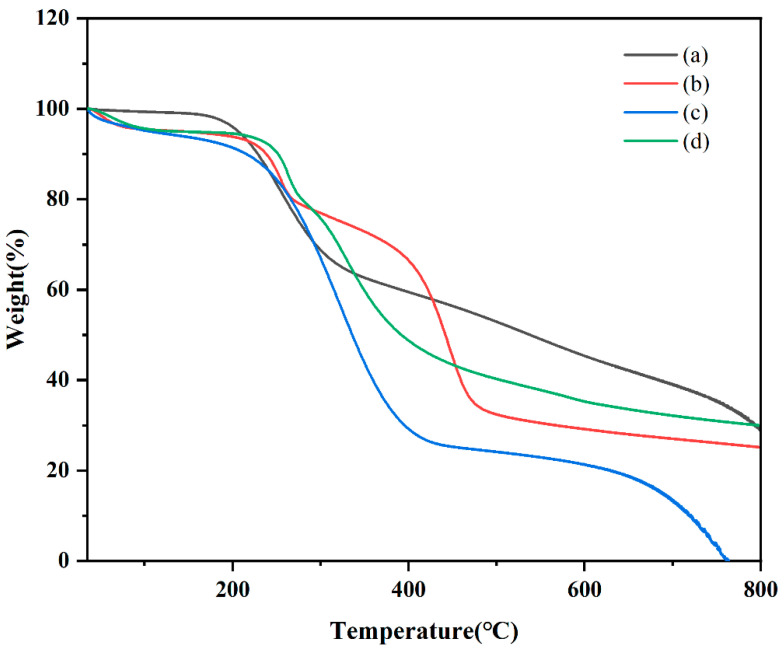
Comparing thermal gravimetric analyzer thermograms: (a) CTC powder; (b) PVPK30-based solid dispersion; (c) HP-β-CD-based solid dispersion; and (d) gelatin-based solid dispersion.

TGA is used to evaluate physical and chemical property adjustments in different materials [37]. In this study, TGA was used to examine CTC, PVPK30, HP-β-CD, gelatin, physical mixtures, and solid dispersions to determine mass loss due to temperature increases. The TGA data are shown in Figure 8 and Figure S3. Although thermograms show the sum of pure component curves, substantial differences can be observed in complex curves [38]. At 400 °C, PVPK30 showed more stability than HP-β-CD and gelatin, with a residual weight of 85.35%. For CTC, decomposition was divided into two periods: the first at 200 °C and the second at 781 °C, with residual weight reduced from 98.639 wt% to 33.806 wt%. In considering unstable CTC physicochemical properties, the TGA curve of the physical mixture showed drug and polymer superposition, with the first decomposition corresponding to drug decomposition and the second corresponding to the polymer. According to the TGA of solid dispersions prepared by medium ball milling, The CTC thermal stability was improved after complexing with polymers, with the polymers decomposing at higher temperatures than the free CTC. It should be noted that the CTC solid dispersion’s thermal stability was less than that for the pure components (PVPK30, HP-β-CD, and gelatin), but the CTC solid dispersion still retained reasonable thermal stability.

#### 2.5.5. Powder X-ray Diffraction (PXRD)

**Figure 9:**
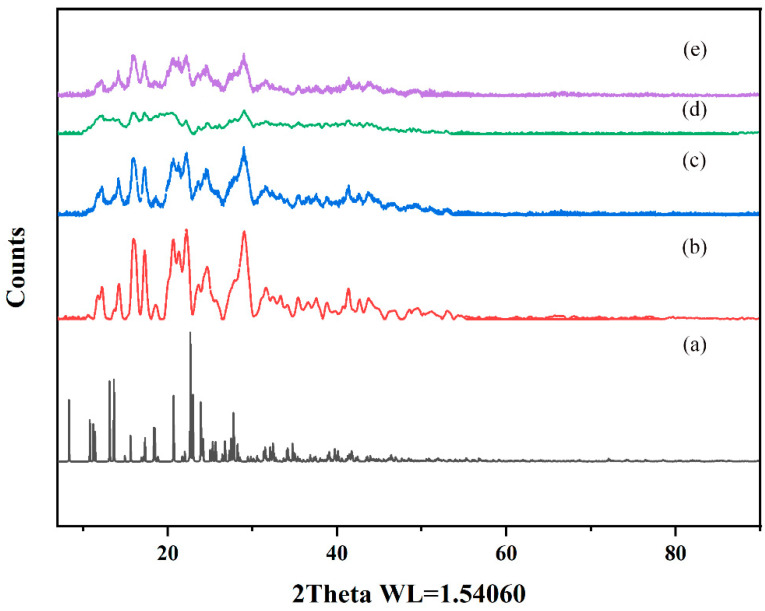
Comparing powder X-ray diffraction patterns: (a) CTC simulated; (b) CTC powder; (c) PVPK30-based solid dispersion; (d) HP-β-CD-based solid dispersion; (e) and gelatin-based solid dispersion.

PXRD effectively characterizes interactions between host and guest molecules and verifies crystal structure properties in mixtures [39]. In this study, the CTC and the solid dispersions were evaluated by PXRD. As shown (Figure 9 and Figure S4), pure CTC powder exhibited obvious characteristic diffraction peaks, indicating a crystal form. The characteristic peaks that appeared coincided highly with the simulated XRD pattern, but the relative intensities differed. This could be due to the preferred orientation of the sample used for diffractogram measurement. PVPK30, HP-β-CD, and gelatin all showed wide peak characteristics indicative of amorphous forms, consistent with previous studies [40,41]. It is worth noting that the PVPK30 and gelatin physical mixtures showed reduced characteristic peak strength for CTC, bearing in mind that both mixtures were composed of CTC/PVPK30 and CTC/gelatin, respectively, with 1:1 and 1:075 weight ratios. Therefore, when compared with pure CTC powder, the CTC peak strength was reduced and weakened. The disappearance of the HP-β-CD physical mixture drug crystals was possibly related to the amorphous properties of cyclodextrin. However, the diffraction patterns of PVPK30- and gelatin-based solid dispersions, prepared by medium ball milling, showed that the characteristic peak intensity was significantly lower than that for the pure CTC powder and the physical mixtures, indicating the presence of amorphous CTC in the solid dispersion. The HP-β-CD-based solid dispersions showed wide peak characteristics, indicating an amorphous CTC form.

#### 2.5.6. Fourier-Transform Infrared Spectroscopy (FT-IR)

**Figure 10:**
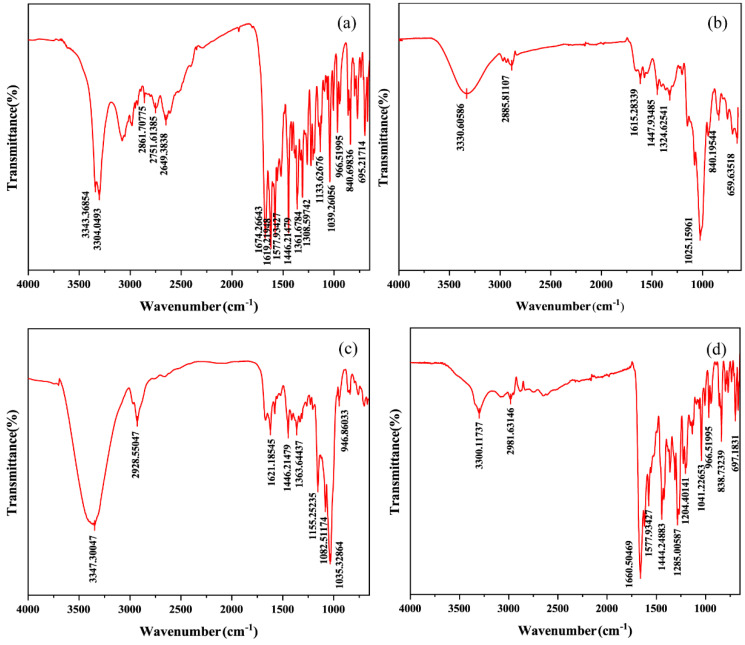
Comparing Fourier infrared spectroscopy spectra: (**a**) CTC powder; (**b**) PVPK30-based solid dispersion; (**c**) HP-β-CD-based solid dispersion; and (**d**) gelatin-based solid dispersion.

FT-IR is used to study the nature and extent of interactions between drugs and carriers and to identify interaction differences between drugs and carriers in physical and media grinding mixtures [39]. Our FT-IR spectra are shown in Figure 10 and Figure S5. The pure CTC spectra showed (Table 2) N-H stretching vibration signals at 3343 and 3304 cm^−1^; C=O double signals at 1674 cm^−1^; aliphatic C-H stretching vibrations at 1446, 1361, and 1308 cm^−1^; and bending vibrations outside the C-H aromatic ring at 840 and 695 cm^−1^. The physical mixtures’ spectral patterns (Figure S5d,e,f) reflected the sum of the FT-IR spectral band positions of PVPK30, HP-β-CD, gelatin, and CTC, with accumulation effects, thus indicating little or no interactions between the drug and polymer in the physical mixtures. However, the spectral patterns for the solid dispersions prepared by medium ball grinding (Figure 10) were significantly different to those for the physical mixtures. In the three solid-dispersion formulations, partial peak displacement occurred, but no new absorption peaks appeared, indicating that CTC had not formed new chemical bonds and that intermolecular interactions occurred between the drug and polymers, leading to hydrogen bond formation.

## 3. Materials and Methods

### 3.1. Materials

The CTC (purity > 85%) and poloxamer were purchased from Beijing Solarbio Technology Co., Ltd. (Beijing, China). The β-CD, HP-β-CD, gelatin, and PVPK30 were obtained from Shanghai Yuanye Bio-Technology Co., Ltd. (Shanghai, China). Polyethylene glycol was purchased from Shanghai Guangnuo Chemical Technology Co., Ltd. (Shanghai, China). Microcrystalline cellulose was purchased from IMCD Co., Ltd. (Shanghai, China). Sodium alginate, polyvinyl alcohol, and ethyl cellulose were purchased from Shanghai Macklin Biochemical Technology Co., Ltd. (Shanghai, China). All the other materials were of analytical reagent grade and used as received. All experiments were conducted using de-ionized water.

### 3.2. Preparing Solid Dispersions by Grinding with CTC-Loaded Medium

The CTC solid dispersions were prepared using the medium grinding method, and the best carrier was determined. At room temperature, CTC was mixed with different PVPK30, gelatin, and HP-β-CD quantities in a small, laboratory-scale medium grinder (Chishun technology development Co., Ltd., Nanjing, China). We used two zirconia grinding tanks to balance the grinding (45 mL); 16 built-in porcelain balls of different sizes, four grinding cycles, 530 revolutions, a grinding time = 30 min, and a combination of forward and reverse rotation were used.

### 3.3. Polymer Effects on CTC Solubility

To select suitable polymers for the CTC solid-dispersion preparations, the CTC solubility was screened using different polymers (gelatin, sodium alginate, HP-β-CD, β-CD, polyvinyl alcohol, PVPK30, poloxamer188, microcrystalline cellulose, ethyl cellulose, and polyethylene glycol). Equal CTC and polymer proportions were added to a 20 mL aqueous solution for 2 h with stirring and then ultrasonicated for 5 min, oscillated at 25 °C (in a water bath) for 7 days, and centrifuged at 12,000 rpm for 15 min, and the supernatants were filtered through 0.22 μm filters. Finally, the CTC concentrations were determined using high-performance liquid chromatography (HPLC).

### 3.4. Polymer Dose Effects on CTC Solubility in Solid Dispersions

PVPK30-based solid dispersions, using CTC-to-PVPK30 weight ratios of 1/0, 1/0.25, 1/0.5, and 1/1, were prepared using the medium grinding method. The HP-β-CD-based solid dispersions were prepared using CTC-and-HP-β-CD weight ratios of 1/0, 1/0.5, 1/1, 1/1.5 and 1/2; and the gelatin-based solid dispersions were prepared using CTC-to-gelatin weight ratios of 1/0, 1/0.25, 1/0.5, 1/0.75, 1/1, and 1/1.5. The ratios were then screened to determine the optimal solid-dispersion proportions. Finally, the CTC concentrations were determined using HPLC.

### 3.5. Stability Study

The stability of the solid dispersions was evaluated by influencing-factor experiments including high temperature, high humidity, and strong light [42]. Precision-weighed amounts of 2.0 g solid-dispersion samples were placed in a plate and placed in a stable oven at 60 °C/75% ± 1%, 40 °C/75% ± 1% (high-temperature, high-humidity test) and 4500 ± 500 l× (light test) for 10 days, respectively. The samples were taken on the fifth and the tenth day to assess changes in the appearance and drug content.

### 3.6. Solubility and Dissolution Studies on CTC-Loaded Solid Dispersions

The dissolution rates of the PVPK30-, HP-β-CD- and gelatin-based solid dispersions (each containing 100 mg CTC) were measured by a dissolution tester at 37 °C and 900 mL distilled water with a rotational speed of 150 rpm/min. Then, 5 mL samples were taken at 5, 10, 15, 30, 45, 60, and 120 min, and the same volume of distilled water added [43]. The samples were then filtered through 0.22 µm microporous filter membranes, and the drug concentrations were determined by HPLC.

### 3.7. HPLC

HPLC was performed using a Waters2489 system (Milford, Massachusetts, USA). The sample solubility and dissolution were analyzed on a C18 column (250 × 4.6 mm, inner diameter = 5 μm; Milford, MA, USA) in a mobile phases of 0.01 M oxalic acid, acetonitrile, and methanol (7:2:1). The chromatographic conditions were flow rate = 1.0 mL/min, temperature = 35 °C, and sample volume = 20 μL. A standard curve was used to calculate the CTC concentrations: Y = 4.9526x − 0.3447, R^2^ = 1. All the experiments were conducted in triplicate.

### 3.8. CTC-Loaded Solid-Dispersion Physicochemical Properties

#### 3.8.1. SEM

The sample powders were analyzed using a JSM-15 SEM (JEOL, Tokyo, Japan) to characterize their morphology. The samples were fixed to a brass column using conductive double-sided tape and kept in a vacuum, using the JFC 40 (JEOL, Tokyo, Japan) ion sputtering coating machine gold plating to improve the electrical conductivity. The samples were then placed into the sample tank of the scanning analyzer, where they were observed and photographed at an accelerated voltage = 10 kV [44].

#### 3.8.2. DSC

Thermal analysis was performed using the MDSC Q999 (T-zero™ DSC Technology, TA Instruments, New Castle, DE, USA) differential scanning calorimeter. The samples were placed in a sealed aluminum pan and heated in a dynamic nitrogen atmosphere at 50 mL/min at a heating rate of 10 °C/min. Among them, the gelatin physical mixture and solid dispersion were heated in the temperature range of 40–350 °C [45], CTC in the temperature range of 28–200, and PVPK30 in the temperature range of 130–600.

#### 3.8.3. TGA

A TGA (Q5000, TA Corporation, New Castle, DE, USA) was used to determine thermal stability. A sample was placed in a platinum sample tray and, in a N2 environment (40 mL/min), the heating rates were increased from 40 °C to 900 °C at 10 °C/min intervals. A TGA curve was generated as the sample mass changed with the increasing temperature [36].

#### 3.8.4. PXRD

PXRD patterns were generated by evaluating the physical state of a sample using a Bruker D40 Advance diffractometer (Bruker, Germany). The samples were analyzed using Cu-Kα radiation (λ = 8.1 A) in the diffraction angle (2θ) range from 7 to 90°. The running data were as follows: scanning step = 0.02° and scanning speed = 1°/s [29]. In addition, the simulated XRD pattern was generated by the vesta3.5.8 software.

#### 3.8.5. FT-IR

The FT-IR spectra were obtained in a Bruker Tensor 27 FT-IR spectrometer (Bruker, Germany). The samples were thoroughly mixed and ground in KBr, after which the powders were compressed to prepare KBr disks, and finally 32 scans were accumulated at 2 cm^−1^ resolution in the 4000–400 cm^−1^ range [46].

#### 3.8.6. PS Distribution Analyses

The average PS and distribution were characterized using laser diffraction. The samples were diluted 100-fold in deionized water and added to a colorimetric dish. The dispersant and refractive indices were set to ensure that the diluted samples were in clear and transparent solutions. Ultimately, the measured sample PS distribution categorized by D90, D50, and D10 is given [47].

### 3.9. Statistical Analyses

The data were expressed as the mean ± SD. The results were analyzed using one-way analysis of variance and Duncan’s multiple range tests in SPSS 20.0 software. The significance level was set at *p* < 0.05.

## 4. Conclusions

In summary, this study successfully prepared a polymer-based solid dispersion using the medium ball milling method, which included hydrophilic polymers such as PVPK30, HP-β-CD, and gelatin to improve the solubility of CTC. And characterization was carried out using the SEM, DSC, TGA, PXRD, FT-IR, and particle size distribution methods. It was found that the physical properties and particle size of the insoluble drug CTC were affected by the type and proportion of the polymer, causing CTC to transition from a crystalline state to an amorphous state, thereby improving the solubility and dissolution rate of the drug. The solubility studies showed that the solubility values for the 1/0.75 PVPK30- (approximately 25 mg/mL), 1/2 HP-β-CD- (approximately 30.8 mg/mL), and 1/1 gelatin-based solid dispersions (approximately 15 mg/mL) were significantly improved when compared with those of pure CTC powder (4 mg/mL). Also, the PVPK30 (57.78%), HP-β-CD (59.95%), and gelatin (60.89%) solid dispersions’ dissolution rates in vitro were significantly higher than those for other CTC preparations. Additionally, the initial dissolution rate of the gelatin-based solid dispersion was significantly higher than that of the PVPK30- and HP-β-CD-based solid dispersions, as the gelatin-based solid dispersion consisted of smaller, more uniform particles bound together to form aggregates. Therefore, the results of this study indicate that the selection of polymer types and ratios is an important and promising strategy for improving the solubility of poorly soluble drugs and ultimately enhancing their bioavailability.

## Figures and Tables

**Table 1:** The influence factor test of CTC-loaded solid dispersion (mean ± SD, *n* = 3).

Groups	Factors	Content (%)		
		0th Day	5th Day	10th Day
PVPK30-based solid dispersion	High light	100	102 ± 0.01	97.53 ± 1.69
	High temperature 60 °C/75% High humility	100	98.2 ± 0.73	97.34 ± 0.77
	High temperature 40 °C/75% High humility	100	98.83 ± 0.14	98.69 ± 0.73
HP-β-CD-based solid dispersion	High light	100	98.45 ± 0.99	96.53 ± 1.03
	High temperature 60 °C/75% High humility	100	98.79 ± 1.55	97.85 ± 2.1
	High temperature 40 °C/75% High humility	100	99.96 ± 0.28	98.78 ± 1.08
Gelatin-based solid dispersion	High light	100	92.5 ± 0.65	91.73 ± 0.31
	High temperature 60 °C/75% High humility	100	94.78 ± 0.68	93.27 ± 0.59
	High temperature 40 °C/75% High humility	100	96.82 ± 1.35	96.02 ± 1.15

**Table 2:** Infrared absorption peak characteristics of functional compounds of infrared spectrometer.

Band Observed/cm^−1^	Assignment
3400~3300	N-H stretching vibration
3000–2800	Saturated C-H bond
1760~1660	C=O scaling
1500~1300	Aliphatic C-H stretching vibrations
900~690	Bending vibrations outside the C-H aromatic ring

## Data Availability

The original contributions presented in this study are included in the article/Supplementary Materials; further inquiries can be directed to the corresponding author/s.

## Supplementary Materials

The following supporting information can be downloaded at https://www.mdpi.com/article/10.3390/ijms251910591/s1: Figure S1: Scanning electron micrographs; Figure S2: Comparing differential scanning calorimetric thermograms; Figure S3: Comparing thermal gravimetric analyzer thermograms; Figure S4: Comparing powder X-ray diffraction patterns; Figure S5: Comparing Fourier-transform infrared spectroscopy spectral patterns.
