# Supplementary material for: Polymers Enhance Chlortetracycline Hydrochloride Solubility
Source: Int J Mol Sci. 2024 Oct 1;25(19):10591. doi: 10.3390/ijms251910591 (PMC11477051; doi:10.3390/ijms251910591)
Supplement: Supplementary file 1 [file ijms-25-10591-s001.zip › ijms-3197127-SI.pdf]

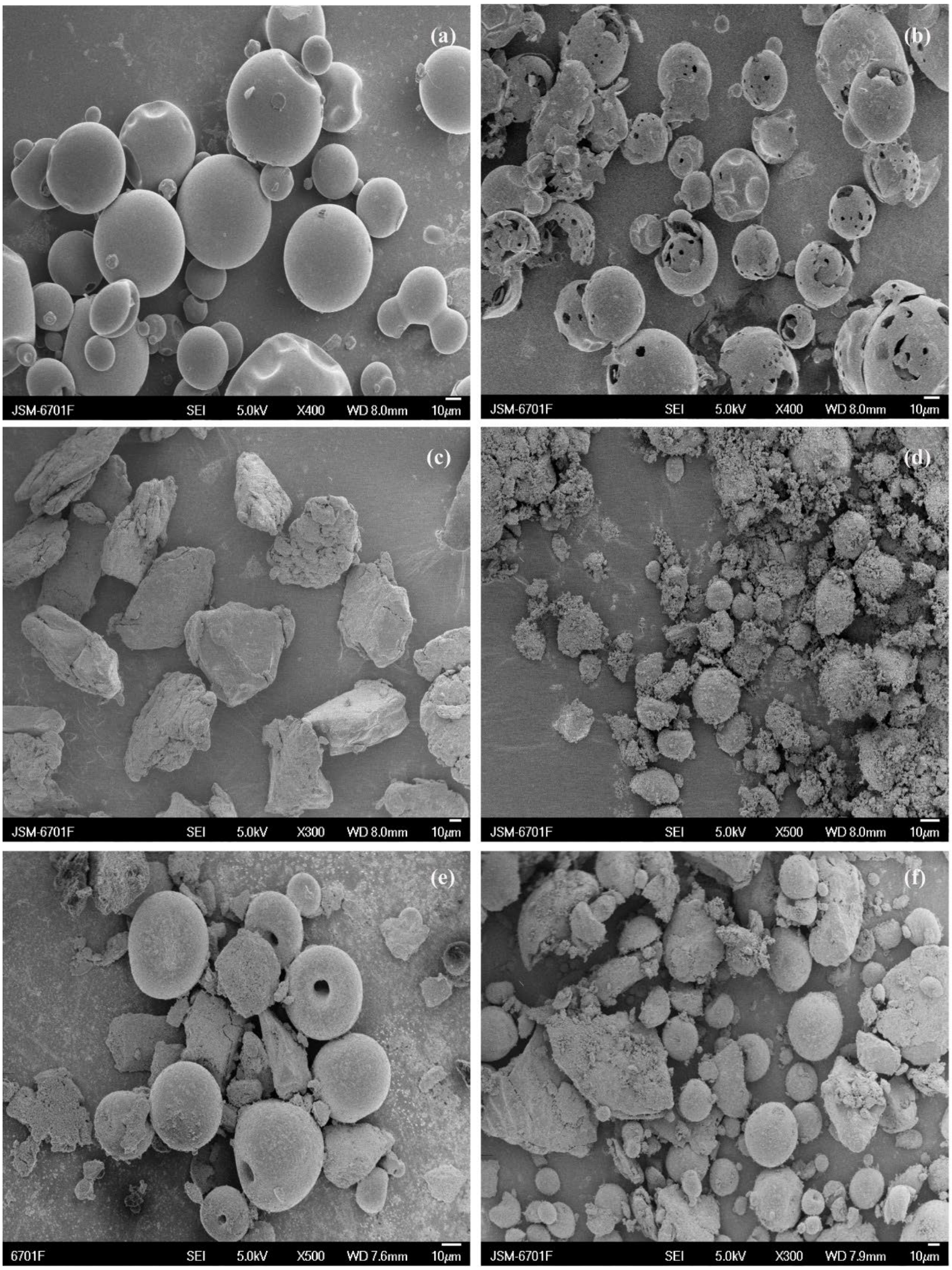

Figure S1. Scanning electron micrographs: (a) PVPK30; (b) HP-β-CD; (c) gelatin; (d) PVPK30 physical mixture; (e) HP-β-CD physical mixture; (f) gelatin physical mixture.

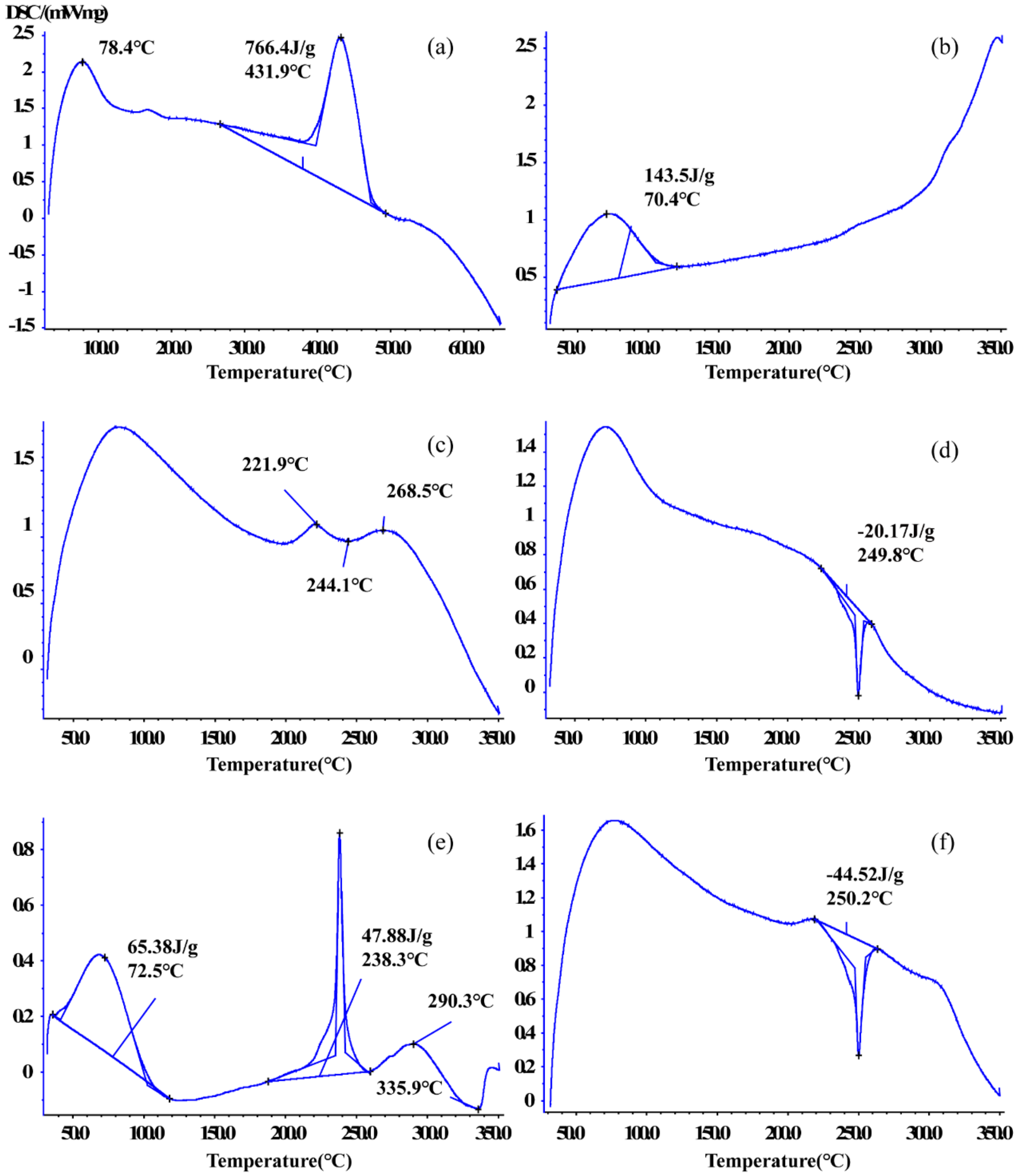

Figure S2. Comparing differential scanning calorimetric thermograms: (a) PVPK30; (b) HP-β-CD; (c) gelatin; (d) PVPK30 physical mixture; (e) HP-β-CD physical mixture; (f) gelatin physical mixture.

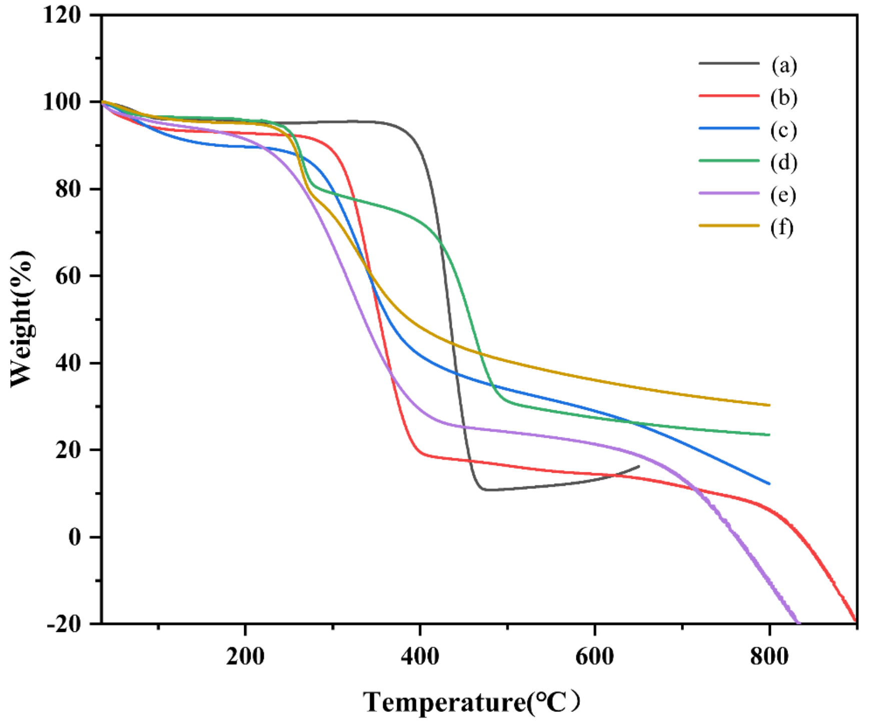

Figure S3. Comparing thermal gravimetric analyzer thermograms: (a) PVPK30; (b) HP-β-CD; (c) gelatin; (d) PVPK30 physical mixture; (e) HP-β-CD physical mixture; and (f) gelatin physical mixture.

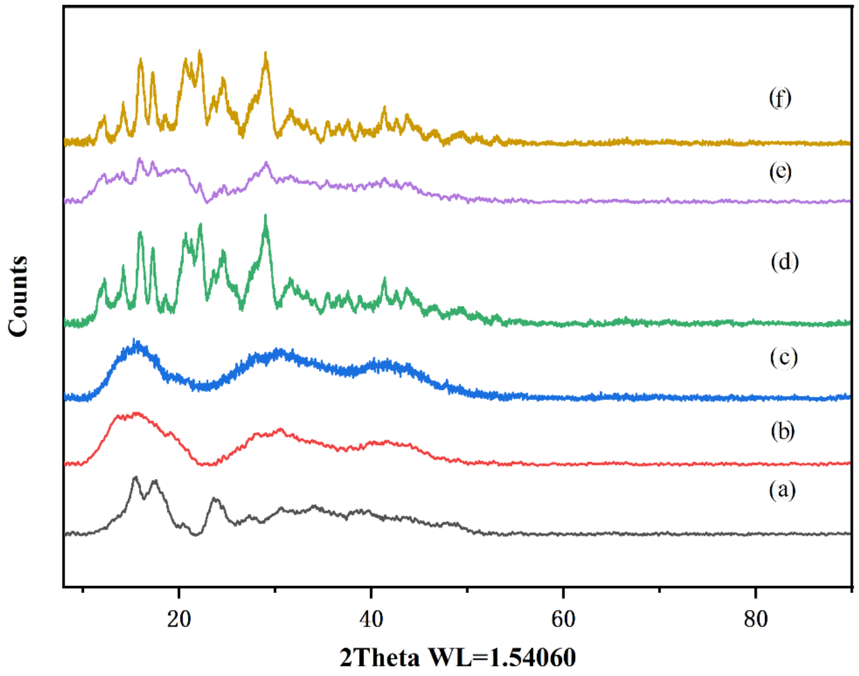

Figure S4. Comparing powder X-ray diffraction patterns: (a) PVPK30; (b) HP-β-CD; (c) gelatin; (d) PVPK30 physical mixture; (e) HP-β-CD physical mixture; and (f) gelatin physical mixture.

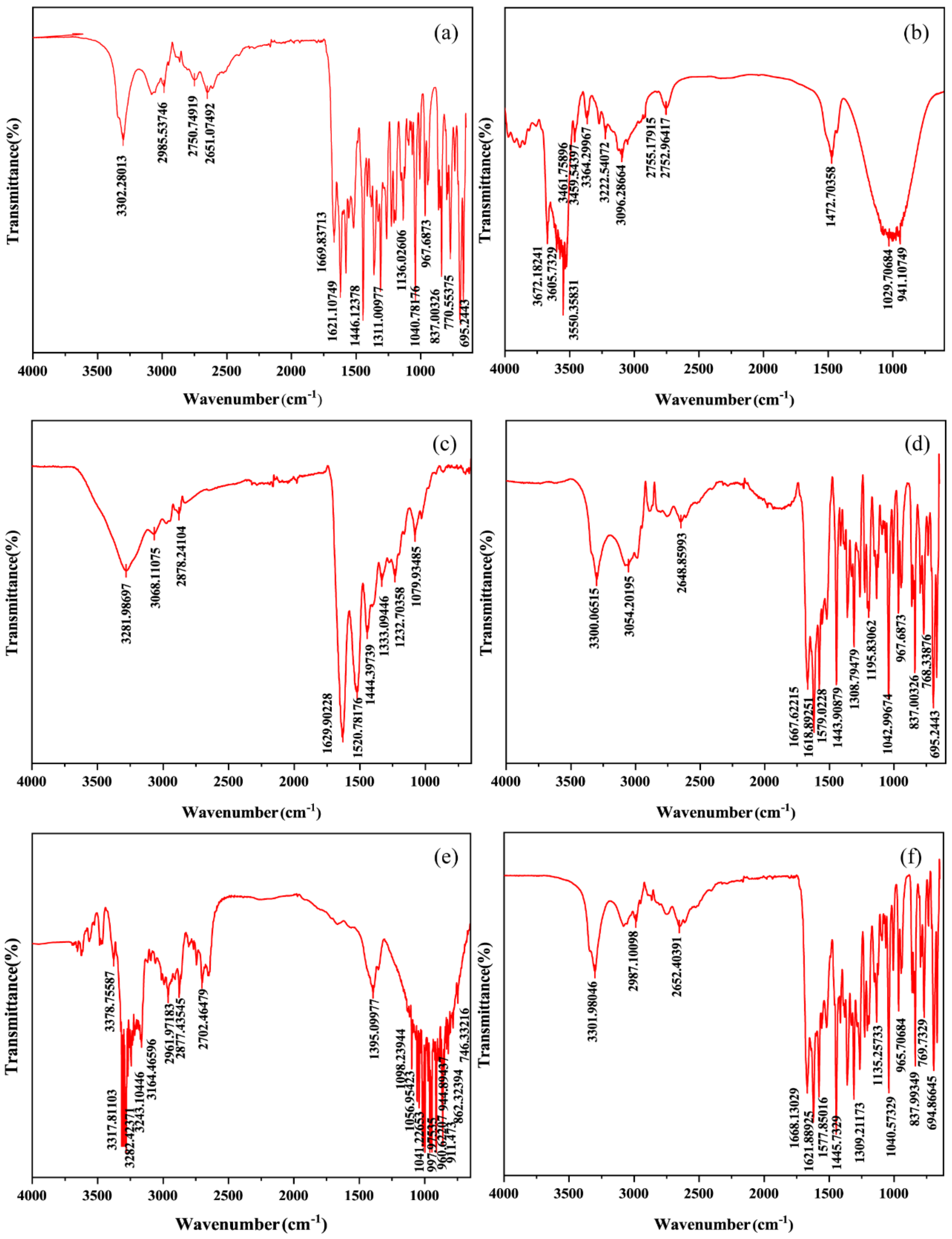

Figure S5. Comparing Fourier transform infrared spectroscopy spectral patterns: (a) PVPK30; (b) HP-β-CD; (c) gelatin; (d) PVPK30 physical mixture; (e) HP-β-CD physical mixture; and (f) gelatin physical mixture.
